# Use of diffusion tensor imaging to assess the impact of normobaric hyperoxia within at-risk pericontusional tissue after traumatic brain injury

**DOI:** 10.1038/jcbfm.2014.123

**Published:** 2014-07-09

**Authors:** Tonny V Veenith, Eleanor L Carter, Julia Grossac, Virginia F Newcombe, Joanne G Outtrim, Sridhar Nallapareddy, Victoria Lupson, Marta M Correia, Marius M Mada, Guy B Williams, David K Menon, Jonathan P Coles

**Affiliations:** 1 Division of Anaesthesia, University of Cambridge, Addenbrooke's Hospital, Cambridge, Cambridgeshire, UK; 2 Wolfson Brain Imaging Centre, Department of Clinical Neurosciences, University of Cambridge, Addenbrooke's Hospital, Cambridge, Cambridgeshire, UK

**Keywords:** contusion, diffusion tensor imaging, normobaric hyperoxia, penumbra, traumatic brain injury

## Abstract

Ischemia and metabolic dysfunction remain important causes of neuronal loss after head injury, and we have shown that normobaric hyperoxia may rescue such metabolic compromise. This study examines the impact of hyperoxia within injured brain using diffusion tensor imaging (DTI). Fourteen patients underwent DTI at baseline and after 1 hour of 80% oxygen. Using the apparent diffusion coefficient (ADC) we assessed the impact of hyperoxia within contusions and a 1 cm border zone of normal appearing pericontusion, and within a rim of perilesional reduced ADC consistent with cytotoxic edema and metabolic compromise. Seven healthy volunteers underwent imaging at 21%, 60%, and 100% oxygen. In volunteers there was no ADC change with hyperoxia, and contusion and pericontusion ADC values were higher than volunteers (*P*<0.01). There was no ADC change after hyperoxia within contusion, but an increase within pericontusion (*P*<0.05). We identified a rim of perilesional cytotoxic edema in 13 patients, and hyperoxia resulted in an ADC increase towards normal (*P*=0.02). We demonstrate that hyperoxia may result in benefit within the perilesional rim of cytotoxic edema. Future studies should address whether a longer period of hyperoxia has a favorable impact on the evolution of tissue injury.

## Introduction

Cerebral ischemia and metabolic dysfunction remain important causes of neuronal loss after traumatic brain injury (TBI).^1^ We have previously used ^15^O positron emission tomography to show that normobaric hyperoxia increases oxygen utilization in ‘at-risk' regions of metabolically compromised tissue, typically in pericontusional regions and white matter.^2^ Such improvements in oxidative metabolism may result through the alleviation of physiologic and metabolic compromise linked to a range of pathophysiological processes. These include classic ischemia, increased diffusion barriers to oxygen delivery associated with microvascular ischemia, and mitochondrial dysfunction.^2, 3^ However, while previous studies demonstrate a consistent effect of hyperoxia in increasing brain tissue oxygen levels, reports of the impact on brain metabolism have been inconsistent, regionally variant, and dependent on the underlying metabolic state of the tissue concerned.^2, 4^ Additional concerns have been raised regarding the potential deleterious effects on pulmonary function and worsening of neuronal injury because of oxidative stress.^5, 6^ Studies within other pathologies such as stroke and myocardial infarction have also shown conflicting evidence of benefit and harm.^7, 8^ Given this background, it is clear that further study of the regional effects of normobaric hyperoxia is warranted before definitive clinical trials of the intervention after TBI.

Diffusion tensor imaging has shown benefit in a variety of neurologic disease states in predicting both local tissue and functional outcome.^9, 10^ Studies after TBI have demonstrated evidence of traumatic axonal injury that is not evident using conventional imaging techniques.^10^ Diffusion tensor imaging (DTI) images dynamic metabolic processes, including cytotoxic edema associated with cellular metabolic failure, and experience in stroke shows that these imaging changes are dynamic and reversible, suggesting that they may be able to image acute treatment effects. We have therefore used DTI to assess the impact of normobaric hyperoxia in this context, and provide data for the planning and design of future therapeutic trials of hyperoxia therapy for patients with head injury.

## Materials and Methods

Ethical approval was obtained from the Cambridgeshire Research Ethics Committee (reference numbers 97/290 and 02/293), and written informed consent, or consultee agreement from next-of-kin where appropriate, were obtained in all cases in accordance with the Declaration of Helsinki.

### Subjects

#### Patients

Fourteen adult patients (12 males and 2 females) with mean (range) age 41 (21 to 70) years with head injury were recruited from the Neurosciences Critical Care Unit, Addenbrooke's Hospital, Cambridge, UK between 2010 and 2012. Patients presented with median (range) postresuscitation Glasgow coma score of 7 (3 to 14), but deteriorated to a Glasgow coma score <8 requiring sedation and ventilation for control of intracranial pressure (ICP; Table 1). Patients were recruited to this imaging study between day 1 and day 9 after injury and underwent imaging while sedated. Patients were excluded from this study if they had suffered a previous TBI, other neurologic disease, or had any contraindication to magnetic resonance imaging All patients were managed by protocol-driven care; which included sedation, paralysis, and ventilation to ensure that ICP<20 mmHg and cerebral perfusion pressure >65 mm Hg were maintained.^2^ Physiologic stability was meticulously ensured during imaging through the titration of fluids and vasoactive agents and the presence of a critical care physician and nurse. Patients who received surgical intervention (cerebrospinal fluid drainage or decompressive craniectomy) or second-tier medical therapies (barbiturate coma or moderate hypothermia (33 to 35 °C)) before imaging are specified in Table 1. No other major changes occurred in the management of patients on the day of study.

After acquisition of baseline diffusion tensor imaging (DTI) at a partial pressure of oxygen (PaO_2_) of approximately 75 to 90 torr (10 to 12 kPa), the fraction of inspired oxygen (FiO_2_) was increased to a maximum of 0.8 to achieve a PaO_2_ of approximately 225 to 260 mm Hg (30 to 35 kPa). After a 60-min period to allow impact of higher PaO_2_ (and by inference, brain pO_2_) levels on cerebral metabolism, repeat DTI was obtained within the same imaging session without moving the patient.

#### Controls

Seven controls (four females and three males) with a mean (range) age of 31 (22 to 42) years were exposed to graded oxygen therapy (21%, 60%, and 100% inspired oxygen) delivered via a venturi mask (Flexicare Medical Limited, Mid Glamorgan, Wales, UK). Diffusion tensor imaging was obtained at each level after an equilibration period of 15 min to assess the impact of oxygen therapy on normal brain.

### Imaging

All subjects were scanned using a 3T Siemens Verio magnetic resonance imaging scanner (Siemens AG, Erlangen, Germany) within the Wolfson Brain Imaging Centre (WBIC), University of Cambridge. During the study period there were no major changes or upgrades to the scanner or software. The sequences obtained were structural sequences including a three-dimensional T1-weighted magnetization prepared rapid gradient echo, fluid-attenuated inversion recovery (FLAIR), gradient echo, susceptibility-weighted images, and dual spin echo (proton density/T2-weighted). The DTI data were acquired using 63 noncollinear directions, *b*=1,000 s/mm^2^ with one volume acquired without diffusion weighting (*b*=0), echo time (TE) 106 ms, repetition time (TR) 11,700 ms, 63 slices, field of view 192 mm × 92 mm, 2 mm^3^ isotropic voxels, and an acquisition time of 13:50 min. All acquired images were reviewed by a specialist neuroradiologist as a part of clinical care.

#### Image processing

Apparent diffusion coefficient (ADC) maps were created using the Oxford Centre for functional magnetic resonance imaging of the brain FSL Diffusion Toolbox.^11^ To aid coregistration, the skull and extracranial soft tissue were stripped from the T1-weighted image using the Brain Extraction Tool of FSL.^12^ The diffusion-weighted data were normalized using a two-step approach. First, T1-weighted images were coregistered to the Montreal Neurological Institute 152 (MNI152) template using the vtkCISG normalized mutual information algorithm. The *b*=0 image was subsequently coregistered to the subject's own T1-weighted image. The transformation matrix normalizing the magnetization prepared rapid gradient echo was then applied to the *b*=0 image. All coregistered and normalized images were visually checked to ensure that they were aligned.

### Region-of-Interest Analysis

#### Standard template in controls

Regions of interest (ROIs) from the Harvard Oxford subcortical and MNI structural probabilistic atlases available within FSL were applied in normalized space.^13^ These included the corpus callosum, midbrain, forceps minor, and forceps major, and bilateral regions covering the frontal, temporal, occipital, and parietal lobes, and the caudate, thalamus, hippocampus, cerebral peduncle, pons, cerebellum, anterior thalamic radiation, superior longitudinal fasciculus, inferior longitudinal fasciculus, cingulum, uncinate fasciculus, and corticospinal tracts. All coregistered images were inspected to ensure that the ROIs were aligned and corresponded to the regions specified. The ROI template was modified by erosion of a single voxel using fslmaths to improve spatial localization and reduce the impact of coregistration, normalization, and partial volume errors. The ADC values for the different ROIs were calculated using in-house software using Matlab (Mathworks, Natick, MA, USA).

#### Lesion-based analysis in patients

**Figure 1:**
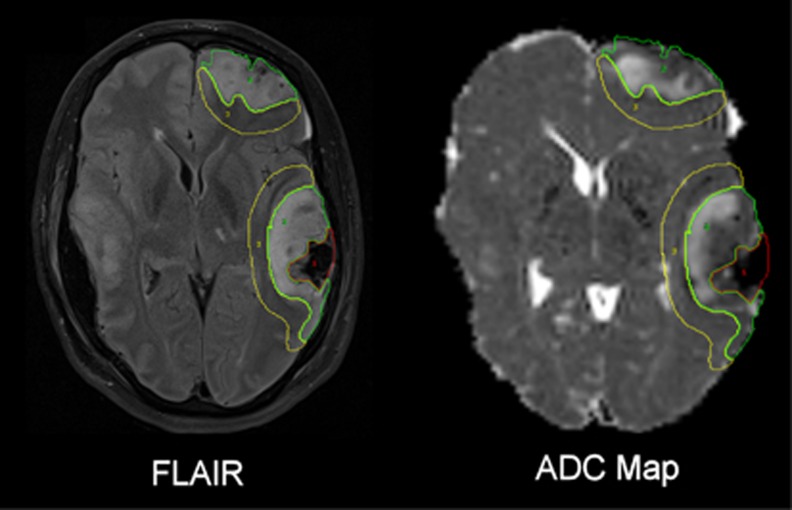
Lesion regions of interest. Fluid-attenuated inversion recovery (FLAIR) and apparent diffusion coefficient (ADC) images with lesion core (red), contusion (green), and perilesion (yellow) identified on a single axial slice.

**Figure 2:**
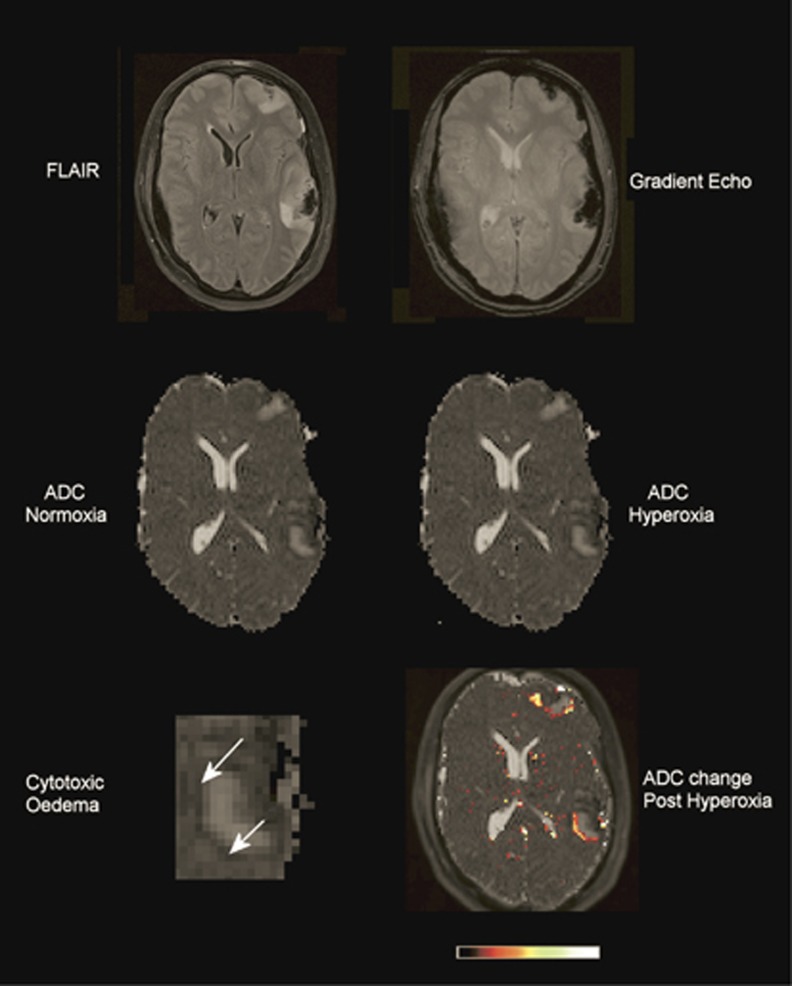
Traumatic penumbra. Fluid-attenuated inversion recovery (FLAIR), gradient echo, and apparent diffusion coefficient (ADC) images at normoxia and hyperoxia demonstrating contusions within the left frontal and temporal parietal regions. These lesions have a hemorrhagic core shown by low signal on the gradient echo corresponding to the presence of blood degradation products, surrounded by a region of ‘vasogenic edema' with high signal on FLAIR and ADC. Around these lesions is a hypointense rim consistent with ‘cytotoxic edema', an example of which is shown at higher magnification and identified by the arrows. The final image has a color map showing the ADC increase calculated from the difference between the ADC images after hyperoxia. This highlights that the increase in ADC occurs predominantly within this border zone immediately surrounding the contusions.

Lesions were defined in native FLAIR space by a single author (JG), and segregated into regions defined as core, contusion, and pericontusion using patient FLAIR, magnetization prepared rapid gradient echo, gradient echo, and susceptibility-weighted images. Lesion core was identified as a region of mixed signal intensity consistent with hemorrhage and necrotic tissue, contusion as an area of high signal on FLAIR, and pericontusion as a 1-cm border zone of tissue surrounding the contusion (Figure 1). Where visible, we also defined a rim of cytotoxic edema (‘traumatic penumbra') on ADC images that we have previously reported around contusions using DTI (Figure 2).^9^ The ROIs were drawn using Analyze 8.5 (Analyze Direct, Lenexa, KS, USA). FLAIR images were coregistered to T1 space using SPM8, and the coregistration matrix subsequently applied to the individual lesion ROIs. For comparison, a comparable region of brain composed of mixed gray and white matter was defined in controls.^14^

#### Statistical analysis

Statistical analyses were conducted using Statview (Version 5, 1998, SAS Institute, Cary, NC, USA). All data are expressed and displayed as mean and standard deviation (s.d.), unless otherwise stated. Individual ROIs were treated independently, as they represented a clinically relevant method of segmenting the brain, with specific location being irrelevant to this analysis. Data were compared using unpaired and paired *t*-tests and analysis of variance. All *P*-values are quoted after Bonferroni corrections (where appropriate), and corrected *P*-values <0.05 were considered significant.

## Results

### Impact of Oxygen Therapy on Diffusion Tensor Imaging in Healthy Volunteers

There was no significant ADC change using the standard template ROI for an increase in the inspired fraction of oxygen (FiO_2_) (*P*>0.99, analysis of variance). The mean (s.d.) ADC was 8.98 × 10^−4^ (1.37 × 10^−4^), 9.21 × 10^−4^ (1.37 × 10^−4^) and 9.20 × 10^−4^ (1.35 × 10^−4^) mm/second for an FiO_2_ of 0.21, 0.6, and 1.0, respectively.

#### Injured brain regions

**Figure 3:**
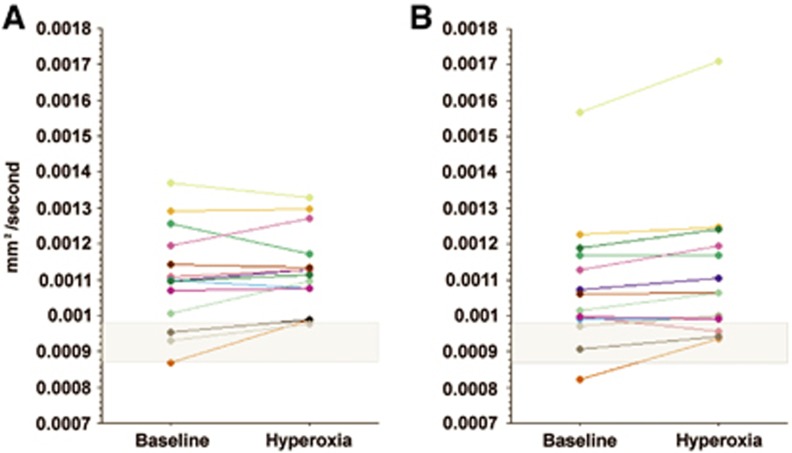
Lesion-based analysis. Apparent diffusion coefficient (ADC) within brain tissue identified as contusion (**A**) and pericontusion (**B**) at baseline and after normobaric hyperoxia. The shaded gray box represents the 95% confidence interval for healthy controls from a region of mixed gray and white matter.

The mean (s.d.) ADC in contusional and pericontusional ROIs was 1.11 × 10^−3^ (1.41 × 10^−4^) and 1.08 × 10^−3^ (1.79 × 10^−4^), respectively, and was significantly higher than controls (9.21 × 10^−4^ (2.78 × 10^−5^, *P*<0.01, analysis of variance with Bonferroni correction)). There was no significant change in ADC after hyperoxia within contusional ROIs (*P*=0.16, paired *t*-test), but an increase within pericontusional ROIs (*P*=0.02, paired *t*-test). One subject with low pericontusional ADC showed an increase to within the normal range. The data are displayed compared with the mixed gray and white matter region from controls (Figure 3).

**Figure 4:**
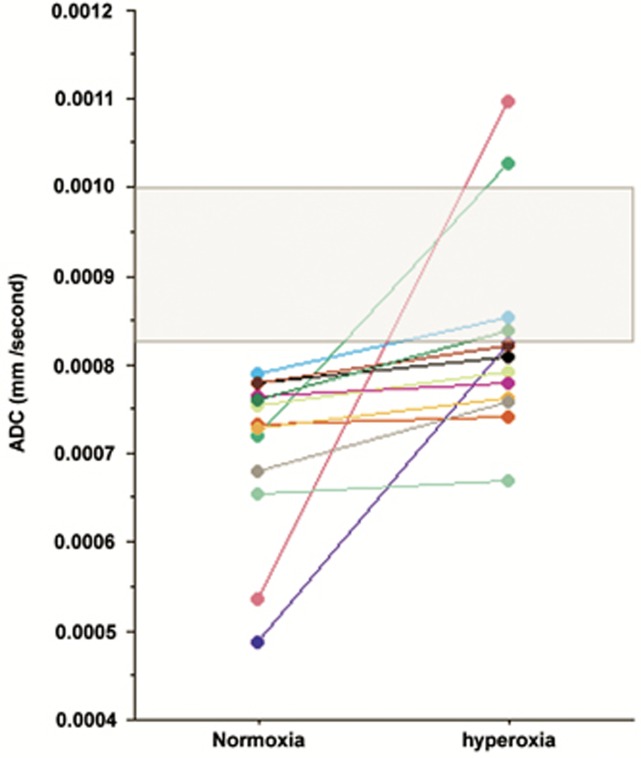
Impact of hyperoxia within traumatic penumbra. Changes in apparent diffusion coefficient (ADC) for the rim of cytotoxic edema surrounding visible brain lesions in 13 subjects. The shaded gray box represents the 99% confidence interval for healthy controls from a region of mixed gray and white matter.

There was a rim of low ADC around brain contusions consistent with cytotoxic edema in 13 subjects with a mean (range) volume of 8 (1 to 20) ml (Figure 2). There was a significant increase in ADC towards the normal range (7.04 × 10^−4^ versus 8.28 × 10^−4^ *P*=0.02, paired *t*-test). The data are displayed compared with a mixed gray and white matter region from controls, and shows that while all subjects demonstrate an increase this is to within or more than the normal range in four subjects (Figure 4).

## Discussion

In this study we used DTI to examine whether an increase in the fraction of inspired oxygen had any beneficial effects within the injured brain. We found no significant change in healthy volunteers and no evidence of benefit within lesion brain identified on structural imaging. The rim of cytotoxic edema that we have previously defined as a region of ‘traumatic penumbra' around brain contusions^9^ demonstrated a significant increase in ADC values towards normal. While an increase in the fraction of inspired oxygen has been reported to increase brain tissue partial pressure of oxygen, reduce microdialysis lactate and lactate pyruvate ratio,^2, 15, 16^ and improve brain metabolism^17, 18^; we show evidence of benefit within ‘at-risk' traumatic penumbral regions of the injured brain. While these data are provisional, they provide a framework to use DTI as an intermediate endpoint to assess the impact of changes in brain oxygenation and metabolism on lesion expansion and local tissue outcome over time within the injured brain. Further studies should address whether there is benefit in using hyperoxia therapy over a longer period of days in patients with head injury.

As oxygen has a known paramagnetic effect^19^ and could, in theory, result in signal change within the MR data we exposed healthy volunteers to three levels of oxygen and conducted DTI at each level. The venturi oxygen masks that we used may not have provided a fixed level of PaO_2_, particularly for oxygen flow rates adjusted to achieve an FiO_2_ of 1.0.^20^ However, at each level of inspired oxygen, PaO_2_ would have been higher in the volunteers and while we do not know the absolute PaO_2_ achieved it would be comparable to the patients who had a maximum FiO_2_ of 0.8 to achieve a PaO_2_ of 225 to 260 mm Hg (30 to 35 kPa). Indeed, the patients all had a degree of lung injury or pathology consistent with trauma and several days of mandatory ventilation. We did not monitor arterial blood gases in the healthy volunteers as the absolute PaO_2_ is not relevant in this case, rather a step increase in oxygenation (baseline, intermediate and maximum) did not result in any consistent change in DTI signal that could explain the result in patients.

Previous studies have demonstrated that patients with an increase in the severity, number, and duration of episodes of tissue hypoxia tend to suffer poor outcome after head injury^21^ and evidence suggests that therapy guided by measurement of brain tissue oxygen levels may be associated with improved outcome.^21, 22^ Interventional studies have demonstrated that an increase in the fraction of inspired oxygen can result in improvements in brain tissue oxygen levels,^15, 23^ and reductions in brain lactate using microdialysis.^2, 16, 23^ While significant, the changes in lactate did not necessarily result in an improvement in oxidative metabolism as the lactate/pyruvate was not consistently lowered.

The effects that result from an improvement in tissue oxygenation are clearly dependent on oxygen delivery and (probably) diffusion gradients in the injured brain.^24^ Pathophysiological derangements within the injured brain are spatially variant and are not limited to regions that appear structurally injured.^1, 3, 25^ Therefore, adequate definition of the effects of hyperoxia across the injured brain demands measurement of regional and global cerebral metabolism using a physiologic imaging technique such as^15^oxygen positron emission tomography. An ^15^O positron emission tomography study showed that ventilation with 100% oxygen in a group of five patients within 24 hours of severe head injury resulted in no change in hemispheric cerebral blood flow or oxygen metabolism (CMRO_2_).^26^ These results are in contrast to a further study that demonstrated that a brief intervention (∼1 hour) of normobaric hyperoxia resulted in an increase of CMRO_2_ within brain regions at the greatest risk of infarction.^2^ This analysis included perilesional and white matter regions of the injured brain. While these data suggest that the impact of hyperoxia may be dependent on the underlying physiologic characteristics of different regions of the injured brain, another study using near-infrared spectroscopy has suggested that short-term therapy with hyperoxia can improve oxygen metabolism within a frontal brain region.^17^

An explanation for these findings comes from postmortem studies showing widespread microvascular occlusion and perivascular edema after TBI, associated with selective neuronal loss.^27, 28^ The relevance of these findings to clinical ischemia is explained by ^15^O positron emission tomography and brain tissue oximetry studies, which show increased vascular to tissue gradients for oxygen tension in the injured brain.^3^ We have previously used DTI to demonstrate contusion expansion,^9^ and that a rim of low ADC consistent with cytotoxic edema is often found surrounding a region of high ADC (vasogenic edema). This rim of hypodensity may characterize a region of microvascular failure resulting in cytotoxic edema, and represent a ‘traumatic penumbra' that may be rescued by effective therapy or be subsumed as the contusion enlarges. Higher brain oxygen levels may overcome diffusion barriers to oxygen delivery, or compensate for mitochondrial dysfunction. Indeed, in regions of low oxygen tension, nitric oxide can competitively inhibit cytochrome oxidase and thereby render mitochondrial respiration dependent on the level of cellular oxygen.^29^ *Ex vivo* studies in clinical and experimental head injury tissue show impaired function in mitochondria (typically <4 hours of injury).^30, 31^ Experimental data also show that mitochondrial ATP production is preserved, and that this is associated with improved cognitive recovery and reduced neuronal cell loss in the hippocampus after injury and treatment with hyperbaric and normoxic hyperoxia.^32^ Experimental data also report that hyperoxia has neuroprotective and antiinflammatory effects within the injured and ischemic brain.^33^ Our clinical data are suggestive of a normalization of ADC values in such regions after a brief period of hyperoxia. However, we have no data on whether such an increase is beneficial in terms of preventing lesion expansion and improving functional outcome. Indeed, in two subjects the increase in ADC was greater than the 95% confidence interval for controls and could reflect tissue injury.

Although the use of high partial pressures of oxygen may be beneficial in a variety of disease states and after brain injury, there may be a relatively narrow margin of safety because of the known toxic effects. The maximum FiO_2_ in this interventional study was limited to 0.8 to reduce potential side effects including alveolar atelectasis and pulmonary injury. However, clinical studies in TBI have used short exposures of normobaric and hyperbaric hyperoxia and failed to demonstrate increased oxidative stress.^34, 35, 36^ While these clinical studies suggest that the use of high concentrations of inspired oxygen in this context may be safe, further studies are required to calculate the risk benefit ratio and determine whether such therapy has a beneficial impact on patient outcome. Such data may permit rational design of future clinical trials.

While evidence of significant changes in brain oxygenation and metabolism^36^ and suggestions that improved outcome may be associated with targeted therapy are encouraging,^21, 37^ a firm recommendation for clinical use of the intervention requires a clinical trial. Previous studies have suggested that hyperoxia therapy in TBI can improve mortality, but not favorable outcome.^38^ A recently published phase II study from Rockswold *et al*^36^ provided valuable evidence of the risks and benefits of hyperoxia therapy over several days. This study compared 60 minutes of hyperbaric hyperoxia (1.5 atmospheres) with 3 hours of 100% oxygen and standard care in a group of 69 patients with severe head injury.^36^ Patients received therapy on three consecutive days starting within 27 hours of injury and demonstrated some evidence of an improvement in cerebral physiology that lasted until the next treatment period. Importantly, there were no signs of pulmonary or cerebral toxicity. A more recent publication from the same group^35^ suggests that a combination of daily hyperbaric (60 minutes at 1.5 atmospheres) followed by 3 hours of normobaric hyperoxia (FiO_2_ 1.0) can result in an increase in favorable outcome.

Despite the promising findings, the studies by Rockswold *et al* do not provide definitive evidence of an improvement in clinical outcome.^35, 36^ Evidence of a change in tissue fate may come from DTI but evidence of improved outcome will require a large clinical trial. Previous studies have shown serial DTI changes in gray and white matter after head injury that represent microstructural injury.^9, 10^ Our study addressed this within the time frame of metabolic changes that we have previously demonstrated with short-term hyperoxia, but was only able to show improvement in DTI parameters within a rim of potentially vulnerable tissue around brain contusions.^9^ However, we can use the data from this study and the recent Rockswold studies to refine the design of a future therapeutic trial of hyperoxia therapy after clinical head injury. In the studies published by Rockswold *et al*, subjects received daily exposure to hyperoxia within the first 4 days after injury, and we have shown that evolution of DTI signal changes within pericontusional tissue is maximal within the first 72 hours.^9, 35, 36, 39^ In our study, 9 of 14 subjects underwent intervention within 72 hours of injury and only two subjects were studied within 24 hours of injury. Previous studies have clearly demonstrated that evidence of ischemia is more evident at earlier time points after injury.^1^ However, derangements in brain metabolism continue for many days after injury,^14, 25^ may be particularly prominent in white matter regions, and have shown evidence of improvement after hyperoxia therapy.^2, 35, 36, 39^ In our study, changes in DTI parameters did not differ between those subjects imaged at earlier compared with later time points (*P*=0.32). While Rockswold *et al*^39^ used daily exposures of normo and hyperbaric hyperoxia, the subjects in our study only underwent an intervention lasting approximately 1 hour using an FiO_2_ of 0.8. Clearly, the partial pressure of oxygen delivered and the duration of exposure may be relevant in determining the impact on outcome but we must balance the potential benefits with the lack of robust safety data beyond 3 days' worth of treatment.

Future studies should seek to confirm whether exposing patients with brain injury to high fractions of inspired oxygen during management of raised ICP over a period of several days is beneficial. This could cover an initial period of 72 hours, but should be reviewed should further management for raised ICP be required. Assessment could focus on DTI progression around cerebral contusions and within white matter regions as an intermediate endpoint, and as a cause of neurocognitive deficits at outcome.^40, 41^ This would require a longitudinal study with imaging at regular intervals and correlation with structural imaging at outcome and neurocognitive assessment at 6 to 12 months after injury. Such evidence would be useful in the design of any future large clinical trial. We have previously reported on the reproducibility of DTI measurements^42^ and found that for ADC the s.d. of ROI measurements was 3.16 × 10^−5^ mm/second. Using such data to calculate sample sizes for interventional and longitudinal clinical studies, we should be able to detect a 10% change in ADC with 95% power at a significance level of 1% within a group of 15 subjects within a single interventional or longitudinal study design.^42^

## Conclusions

Previous studies have suggested that cerebral metabolism can be improved through an increase in the fraction of inspired oxygen. Using DTI we demonstrate that a short interval of normobaric hyperoxia may result in benefit within the rim of cytotoxic edema around brain contusions. Future longitudinal studies should address whether a longer period of hyperoxia therapy during the time that patients require critical care management of raised ICP has a favorable impact on the evolution of tissue injury. Such data would help inform the design of future clinical trials of targeted oxygen therapy for patients with head injury.

## Figures and Tables

**Table 1:** Patient characteristics

***Subject***	***Age***	***Sex***	***Mechanism***	***Injury***	***GCS***	***Marshall score***	***APACHE II***	***ISS***	***Neurosurgery***	***Second-tier therapies***	***Days to MRI***	***GOS***
1	53	M	RTA	Multiple contusions and DAI	4	NEML	17	34	—		4	MD
2	34	M	RTA	tSAH, SDH and IVH	4	NEML	21	20	EVD		3	VS
3	34	M	Assault	Multiple contusions	8	EML	25	16	DC		3	SD
4	21	M	RTA	Multiple contusions	10	NEML	21	50	—	Hypothermia	2	MD
5	31	M	RTA	Multiple SDH	6	EML	17	29	DC		1	MD
6	29	M	Assault	Multiple contusions	10	EML	17	16	DC, EVD	Hypothermia	2	GR
7	58	M	Fall	Multiple contusions	10	NEML	20	34	—	—	4	GR
8	26	M	RTA	SDH and Multiple contusions	3	NEML	17	75	—		3	MD
9	28	M	Assault	SDH and EDH	12	EML	24	36	DC		3	GR
10	61	M	Fall	Multiple contusions	5	NEML	22	75	—		9	Not available
11	60	M	Fall	Multiple contusions	14	NEML	8	34	—	—	3	MD
12	31	F	Fall	Multiple contusions	3	EML	25	75	DC	Hypothermia	4	VS
13	70	F	RTA	Multiple contusions	3	NEML	21	34	—		1	GR
14	27	M	RTA	Multiple contusions	7	NEML	16	25	—		4	GR

DAI, diffuse axonal injury; DC, decompressive craniectomy; EDH, extradural hemorrhage; EML, evacuated mass lesion; EVD external ventricular drain; F, female; GCS, Glasgow coma score; GOS, Glasgow outcome score; GR, good recovery; IVH, intraventricular hemorrhage; M, male; MD, moderate disability; MRI, magnetic resonance imaging; NEML, non evacuated mass lesion; RTA, road traffic accident; SD, severe disability; SDH, subdural hemorrhage; tSAH, traumatic subarachnoid hemorrhage; VS, vegetative state.

## Notes

The funders had no role in study design, data collection and analyses, decision to publish, or preparation of the manuscript.
